# Enhanced Osteogenicity of Bioactive Composites with Biomimetic Treatment

**DOI:** 10.1155/2014/207676

**Published:** 2014-04-09

**Authors:** Ville V. Meretoja, Teemu Tirri, Minna Malin, Jukka V. Seppälä, Timo O. Närhi

**Affiliations:** ^1^Department of Prosthetic Dentistry, University of Turku, Lemminkäisenkatu 2, 20520 Turku, Finland; ^2^Turku Clinical Biomaterials Centre, University of Turku, Itäinen Pitkäkatu 4B, 20520 Turku, Finland; ^3^Polymer Technology, School of Chemical Technology, Aalto University, P.O. Box 16100, Aalto, 00076 Espoo, Finland; ^4^Clinic of Oral Diseases, Turku University Central Hospital, Lemminkäisenkatu 2, 20520 Turku, Finland

## Abstract

*Purpose*. This study aimed to explore if initiation of biomimetic apatite nucleation can be used to enhance osteoblast response to biodegradable tissue regeneration composite membranes. *Materials and Methods*. Bioactive thermoplastic composites consisting of poly(**ε**-caprolactone/DL-lactide) and bioactive glass (BAG) were prepared at different stages of biomimetic calcium phosphate deposition by immersion in simulated body fluid (SBF). The modulation of the BAG dissolution and the osteogenic response of rat mesenchymal stem cells (MSCs) were analyzed. *Results*. SBF treatment resulted in a gradual calcium phosphate deposition on the composites and decreased BAG reactivity in the subsequent cell cultures. Untreated composites and composites covered by thick calcium phosphate layer (14 days in SBF) expedited MSC mineralization in comparison to neat polymers without BAG, whereas other osteogenic markers—alkaline phosphatase activity, bone sialoprotein, and osteocalcin expression—were initially decreased. In contrast, surfaces with only small calcium phosphate aggregates (five days in SBF) had similar early response than neat polymers but still demonstrated enhanced mineralization. *Conclusion*. A short biomimetic treatment enhances osteoblast response to bioactive composite membranes.

## 1. Introduction

Bioactive glasses (BAGs) are a group of silica-based materials used as bone substitutes, typically in the form of powders and rigid monoliths. Regardless of their specific composition, BAGs form an apatite-like surface layer in physiological conditions, which is the prerequisite for their bone bonding ability [1]. Furthermore, the soluble BAG dissolution products may be equally important for bone regeneration [2]. Although their osteopromotive properties are well known, clinical use of BAGs has been restricted to the replacement of bony parts under low loads and as bone fillers [3, 4]. Synthetic polymer BAG composite materials may achieve beneficial handling and mechanical properties, thus increasing the range of possible clinical applications. Both biodegradable and nondegradable bioactive composites have been developed, for example, tissue engineering scaffolds [5], bone cements [6], and even load-bearing implants [7].

Thermoplastic bioactive composite consisting of poly(*ε*-caprolactone-co-D,L-lactide), P (CL/DLLA), and BAG granules has shown interesting properties [8]. The composite can be plasticized by heating it up to the melting temperature of 50°C, after which it remains moldable for some minutes in ambient temperature. In simulated body fluid (SBF), a biomimetic mineral layer is formed on the material resembling the function of BAGs [9]. Such prefabricated biomimetic surfaces are further proposed to enhance bone tissue response to implanted materials [10].

The aim of this study was to evaluate if SBF treatment can enhance* in vitro* osteogenesis on the composite membranes and how this response is modulated by the treatment time.

## 2. Materials and Methods

### 2.1. Culture Substrates

P(CL/DLLA) was synthesized from the corresponding monomers in a ring-opening polymerization. Monomer ratio (mol/mol) in feed was 96/4 (CL/DL-LA). Glycerol was used as a coinitiator and stannous octoate as a catalyst [8]. Bioactive thermoplastic composite was prepared by blending BAG granules (90–315 *μ*m; BonAlive Biomaterials Ltd., Turku, Finland) into the copolymer in a batch mixer (Brabender W50EH, Germany; 100°C, 60 rpm, 5 min). The substrate materials were compressed at 80°C into discoid specimens. Two kinds of discs (*Ø*10 × 2 mm) were prepared: copolymer without BAG granules (P0) and composite with 60 wt-% of BAG (C0).

### 2.2. Immersion in SBF

SBF was prepared by dissolving NaCl, NaHCO_3_, KCl, K_2_HPO_4_·3H_2_O, MgCl_2_·6H_2_O, CaCl_2_·2H_2_O, and Na_2_SO_4_ into deionized water. The solution was buffered to physiological pH 7.4 at 37°C with tris(hydroxymethyl)aminomethane and hydrochloric acid. The ion composition of the SBF corresponds to inorganic portion of human blood plasma [11]. Composite substrates were sterilized in 70% ethanol and were subsequently washed with deionized water. The sterilized specimens with 4 mL of SBF were closed in test tubes and incubated in a shaking water bath at 37°C for 5 and 14 days (substrates C5 and C14). Substrates used in the cell culture experiments are described in Table 1.

### 2.3. Cell Cultures

Bone marrow derived mesenchymal stem cells (MSCs) were harvested from young male adult Sprague-Dawley rats. Retrieved femurs were wiped with 70% alcohol and immersed twice in DMEM with Pen Strep antibiotics (Gibco BRL, Life Technologies BV, The Netherlands). The condyles were cut off and bone marrow was flushed out using culture medium supplemented with 10% fetal bovine serum (FBS; Gibco). The resulting suspension was passed through a 22-gauge needle and plated in culture flasks. After 7 days of primary culture, cells were trypsinized and resuspended in osteogenic culture medium (*α*-MEM (Sigma Chemical Co., MO), antibiotics, 15% FBS, 50 *μ*g/mL ascorbic acid (Sigma), 5 mM Na-*β*-glycerophosphate (Merck, Germany), and 10 nM dexamethasone (Sigma)).

SBF-treated and untreated substrates were sterilized as before and subsequently immersed once in phosphate buffered saline (PBS) and once in culture medium at 37°C, for one hour each. Cell suspension was added to the substrates at a density of 10 000 cells/cm^2^ and allowed to adhere overnight. After seeding, osteoblast culture was continued for two weeks in 24-well plates with medium replacement every two to three days. The whole cell culture process was repeated in two independent runs.

### 2.4. Ion Concentration Analysis

Aliquots (*n* = 4) of spent culture medium and SBF were taken to monitor evolution of silica, calcium, and phosphate concentrations. Colorimetric measurements of silica and orthophosphate were based on molybdenum blue method. Silicomolybdate complex was reduced with a mixture of 1-amino-2-naphthol-4-sulphonic acid and sulphite, and tartaric acid was used to eliminate interference from phosphate [12]. The antimony phosphomolybdate complex was reduced with ascorbic acid [13]. Calcium concentrations were determined using* ortho*-cresolphthalein complexone (OCPC) method [14]. The assay reagent consisted of OCPC with 8-hydroxyquinol in an ethanolamine/boric acid buffer. Absorbances (820 nm for silica, 700 nm for phosphate, and 560 nm for calcium) were measured using either UV-1601 spectrophotometer (Shimadzu, Australia) or Multiskan MS ELISA plate reader (Labsystems, Finland).

### 2.5. Cell Activity

Proliferation of cells was determined using AlamarBlue assay (BioSource International, CA) in a colorimetric format. Culture substrates (*n* = 4) were washed in PBS and placed into clean 24 wells. Fresh culture medium with 10% assay reagent was added, and after three-hour incubation absorbance values of the medium were read at 560 nm and 595 nm using the ELISA plate reader. Measured absorbances were used to calculate the reduction of assay reagent, and the cell activities were normalized with respect to those on P0 on day 1.

### 2.6. Alkaline Phosphatase Activity

Culture substrates (*n* = 4) were washed in PBS and placed into clean 24 wells containing 0.5 mL of lysis buffer (50 mM Tris-HCl, 0.1% Triton X-100, 0.9% NaCl, pH 7.6). The cells were lysed with freezing-thawing method, and the substrates were washed with 0.5 mL of buffer. The released amount of total protein and alkaline phosphatase (ALP) activity were measured from supernatant diluted with 0.9% NaCl as needed.

Protein concentrations were measured by pipetting equal amounts of supernatant and micro-BCA working reagent (Pierce, IL) to three replicate microtiter wells, followed by a three-hour incubation at 37°C. Absorbances were recorded at 560 nm using the ELISA plate reader and amounts of protein were read from a bovine serum albumin standard curve.

To measure ALP activity, 50 *μ*L of supernatant was transferred to three replicate microtiter wells and 200 *μ*L of* para*-nitrophenylphosphate substrate solution (Sigma A3469) was added. After one hour incubation at 37°C, 50 *μ*L of a 3 M NaOH solution was added into each well to stop the enzymatic reaction. Absorbances were recorded at 405 nm using the ELISA plate reader and amounts of converted substrate were read from a* para*-nitrophenol standard curve. The measured ALP activities were normalized in relation to the amounts of protein in each respective sample.

### 2.7. RT-PCR

PolyA mRNA was isolated using QuickPick mRNA magnetic beads (Bio-Nobile, Finland). Four replicate RNA pools from each substrate type were reverse transcribed with random hexamer primers using GeneAmp Gold RNA PCR Reagent Kit (Applied Biosystems). The resultant first-strand cDNA was analyzed in duplicate PCR reactions using iQ Supermix kit (Bio-Rad Laboratories) and FAM-labeled TaqMan Gene Expression Assays (Applied Biosystems) for bone sialoprotein (BSP; Rn00561414_m1), osteocalcin (OC; Rn00566386_g1), and glyceraldehyde-3-phosphate dehydrogenase (GAPDH, a control gene; Rn99999916_s1). PCRs were carried out using an iCycler iQ real-time PCR detection system with software version 3.1 (Bio-Rad Laboratories). The following cycling conditions were used: 95°C/5 min; 40 cycles of 95°C/20 s, 60°C/60 s. Target gene expression was first normalized to the expression of the housekeeping gene GAPDH in the same sample (ΔCt) and then converted to a fold ratio as compared to the average baseline expression of that target gene measured in P0 group at 7 days (ΔΔCt). Finally, the 2^−ΔΔCt^ method was used to convert normalized gene expression levels to fold differences and statistics were calculated on these values [15].

### 2.8. Scanning Electron Microscopy

After cell activity measurements, the analyzed specimens were washed in PBS and fixed with 2% glutardialdehyde in a 100 mM cacodylic acid buffer pH 7.4. The specimens were subsequently dried in a rising alcohol series and coated with carbon evaporation for scanning electron microscopy (SEM; JSM-5500, Jeol, Japan) and energy dispersive spectrometer (EDS; PGT, NJ) analysis. SEM/EDS was also used to study noncultured substrate surfaces.

### 2.9. Statistics

Results are presented as means ± standard deviations. Statistical analyses were performed using the SPSS v.14.0 software package (SPSS Inc.). Independent samples *t*-test was used for ion concentrations to analyze deviations from the initial state (P0 at 4 days). RT-PCR data were analyzed using the Kruskal-Wallis test followed by the Mann-Whitney *U* test, whereas all other data was analyzed with one-way ANOVA followed by the Tukey's post hoc test. For alkaline phosphatase activities logarithmic transformation was applied. Differences were considered significant at 95% level.

## 3. Results

### 3.1. Bioactive Glass Dissolution

**Figure 1:**
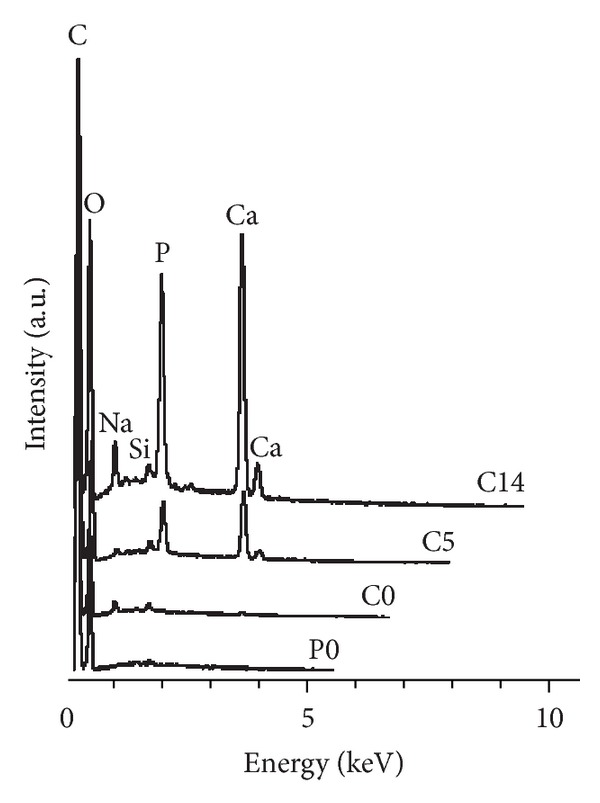
EDS analysis of the substrate surfaces. Nontreated polymer and composite surfaces did not contain Ca and P, whereas the amount of CaP mineral deposition in SBF increased as a function of time. Ca/P ratio in the formed mineral was ~1.3, and some Na and Si were also present on the composite surfaces.

**Figure 2:**
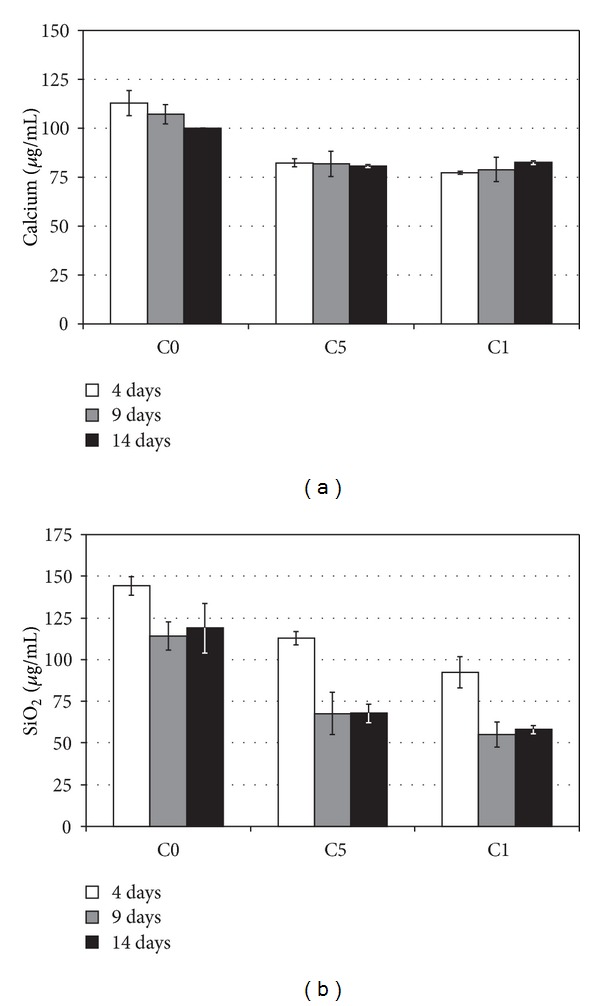
Calcium (a) and silica (b) concentrations of the cell culture medium at days 4, 9, and 14. SBF treatment decreased silica concentrations in the C5 and C14 medium by 50%.

During the immersion of composite substrates (C5 and C14) the SBF became saturated by dissolving silica, and calcium phosphate (CaP) precipitation depleted the majority of phosphorus already in 5 days. Only minor changes in ion concentrations occurred between 5 and 14 days. However, the bioactive glass dissolution and concomitant CaP precipitation continued and the amount of mineral was greater on C14 than C5 substrates. EDS analysis indicated that Ca/P ratio in the formed mineral was ~1.3, and some Na and Si were also present on the substrate surfaces (Figure 1). None of the substrates showed mineral formation when placed into cell-free culture medium. SBF treatment decreased silica concentrations in the medium by roughly 50% and calcium release from composite substrates was eliminated (Figures 2(a) and 2(b)).

### 3.2. Cell Proliferation

**Figure 3:**
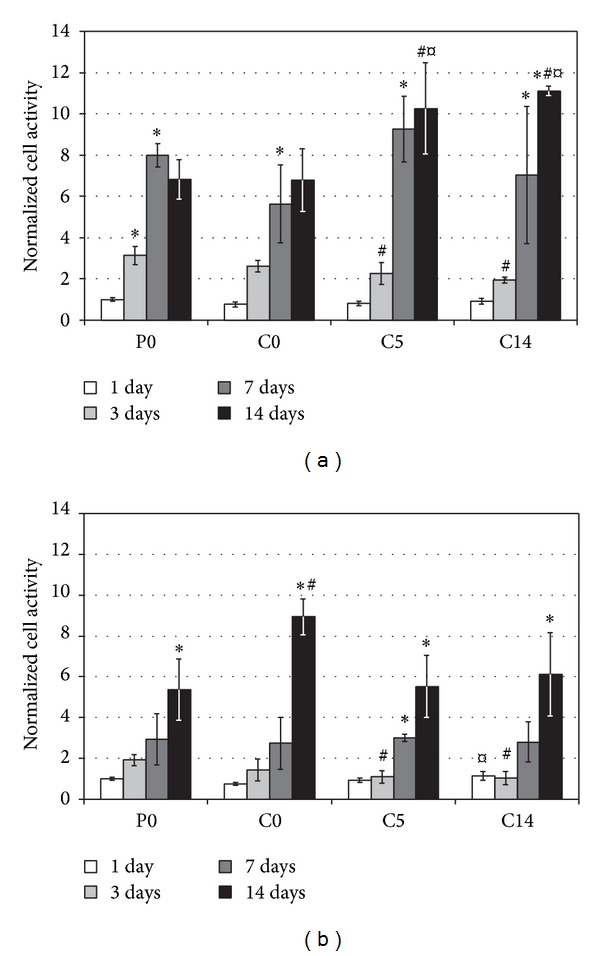
Cell activities in the first (a) and the second (b) osteoblast cultures. The reduction of AlamarBlue reagent with P0 substrates at 1 day was set to 1. Results are presented as mean ± SD with *n* = 4. #, ¤, and ∗ denote statistically significant difference to the corresponding P0 and C0 cultures and to the previous time point, respectively (*P* < 0.05).

**Figure 4:**
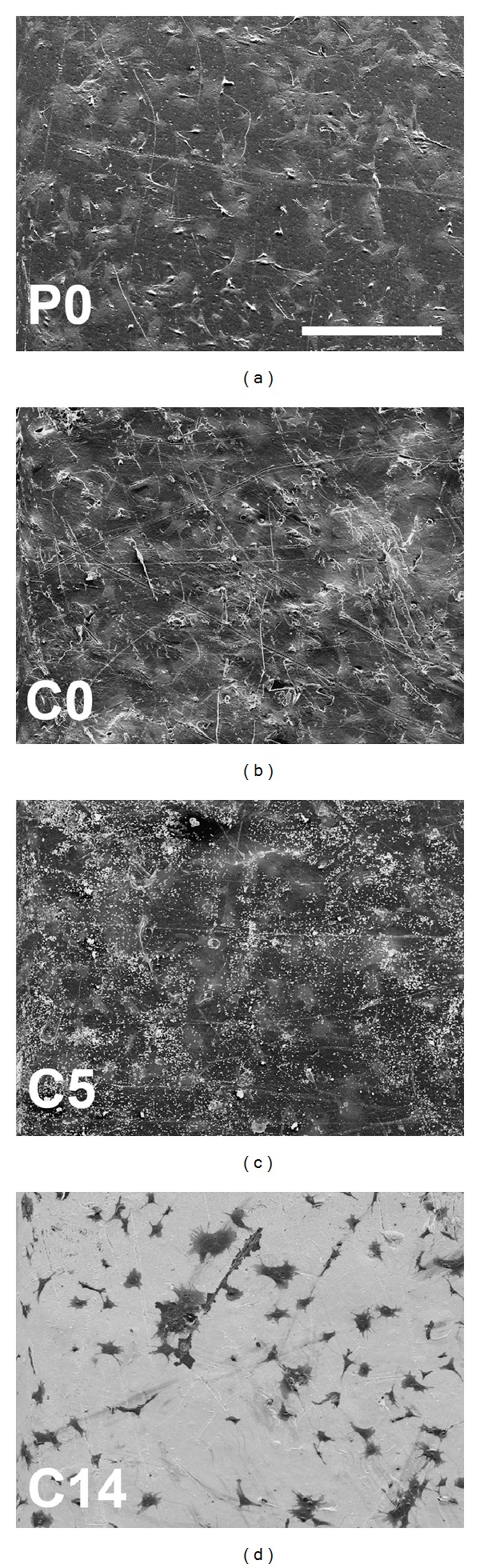
SEM analysis of the substrate surfaces after three days of culture. Cells were spreading over all the substrate surfaces, but the amount of cells seemed to be higher on nontreated than SBF-treated substrates. Small aggregates of CaP mineral were present throughout the C5 surfaces (white dots), whereas C14 substrates were covered by thick mineral layer. Scale bar = 400 *μ*m.

In the first culture, cells proliferated for one week and no further increase in the measured activities was observed, except for C14 substrates. In contrast, all the substrates showed increasing activities up to two weeks in the second culture (Figures 3(a) and 3(b)). Similar cell adhesion after one day was observed with all substrates. The fastest onset of proliferation seemed to occur on neat polymer. Cell activities after three days of both cultures were significantly higher on P0 than on C5 and C14, and the difference was also visible in scanning electron micrographs (Figure 4).

### 3.3. Alkaline Phosphatase Activity

**Figure 5:**
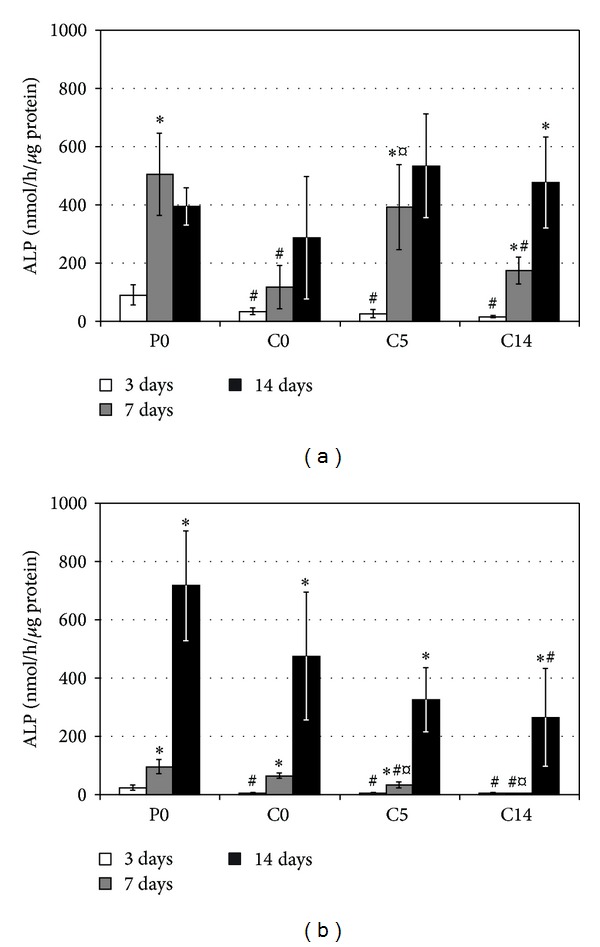
Alkaline phosphatase activities in the first (a) and the second (b) osteoblast cultures. Results are presented as mean ± SD with *n* = 4. #, ¤, and ∗ denote statistically significant difference to the corresponding P0 and C0 cultures and to the previous time point, respectively (*P* < 0.05).

In the first culture, ALP activities with P0 and C5 reached their maximum values already after one week, whereas activities with C0 and C14 increased until two weeks. In the second culture, initial ALP activities were significantly lower than in the first one, and the activities increased until two weeks with all substrates (Figures 5(a) and 5(b)). Furthermore, initial ALP activities with P0 were higher than with composites in both cultures. No other consistent differences between the substrate types were observed.

### 3.4. Mineralization

**Figure 6:**
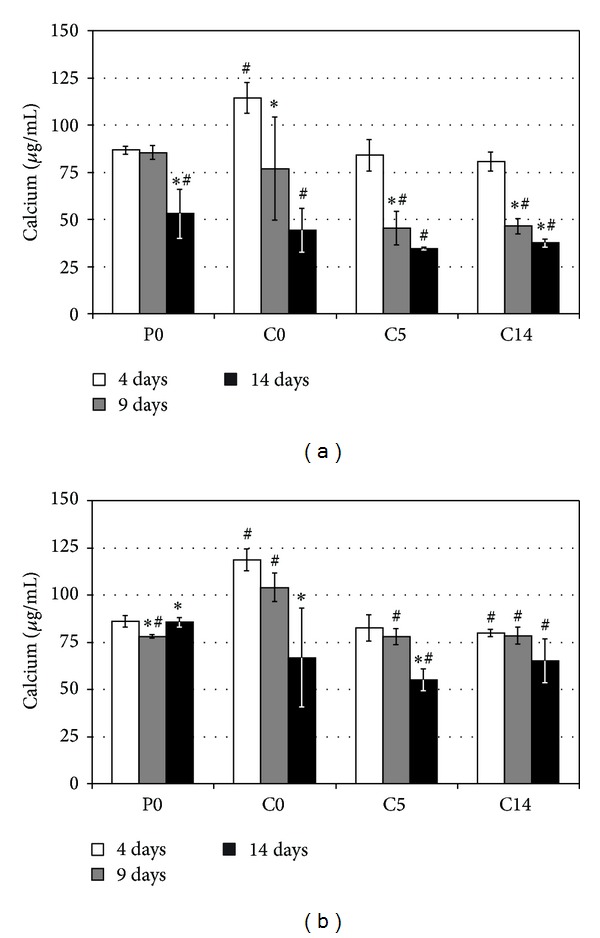
Evolution of calcium concentrations in the first (a) and the second (b) osteoblast cultures. Results are presented as mean ± SD with *n* = 4. # and ∗ denote statistically significant difference to the baseline P0 value and to the previous time point, respectively (*P* < 0.05).

Mineralization, indicated by calcium depletion from the culture medium, proceeded more quickly in the first than in the second culture (Figures 6(a) and 6(b)). However, the SBF-treated composites C5 and C14 were the first and the polymer P0 the last substrates to show calcium precipitation in both cases. Untreated composites C0 showed great variation in the onset of mineralization between individual specimens. Some specimens started to mineralize as early as the SBF-treated ones, whereas other specimens mineralized several days later.

### 3.5. RT-PCR

**Figure 7:**
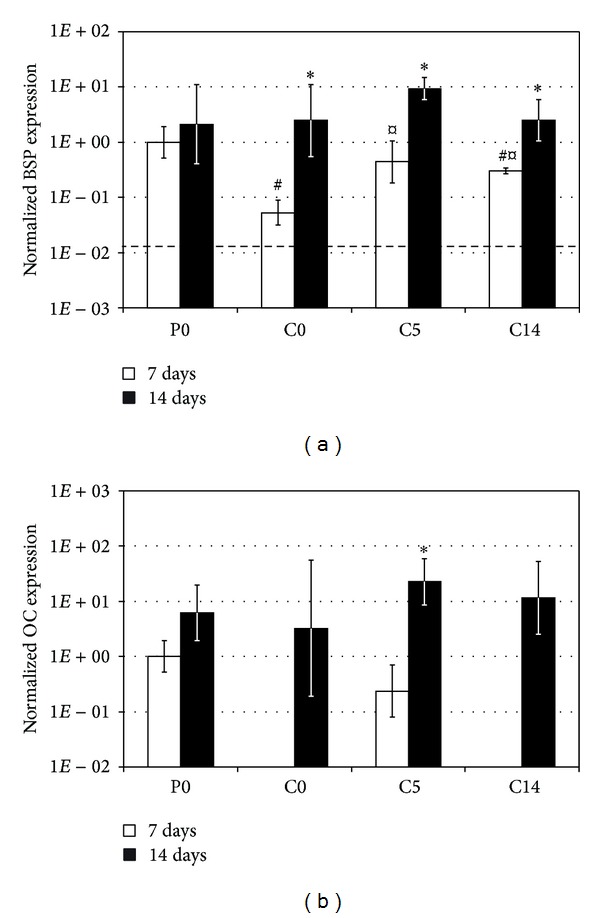
Normalized bone sialoprotein (a) and osteocalcin (b) gene expression levels in the second osteoblast culture. Results are presented as mean ± SD with *n* = 4. #, ¤, and ∗ denote statistically significant difference to the corresponding P0 and C0 cultures and to the previous time point, respectively (*P* < 0.05). The dashed line in (a) represents the BSP expression level of the original cell stock.

The cell stocks used to seed the substrates exhibited low BSP expression (normalized expression level ~0.1 and 0.01 in the first and the second culture, resp.), and OC expression level was below the detection limit (<0.001). Evolution of gene expression was analyzed only in the second osteoblast culture, and the results are summarized in Figures 7(a) and 7(b). After one week of culture, highest BSP and OC levels were observed with P0 and C5 substrates. In contrast, only modest osteogenic induction was observed with C0 substrates. BSP expression was lower than with other substrate types (*P* < 0.01), and OC was still undetectable. C14 substrates also had undetectable OC expression, but BSP level was similar to that with P0 and C5. Osteogenic differentiation progressed during the second week of culture with all substrate types. Both BSP and OC expression levels increased, and there were no statistically significant differences between the substrates at that time.

## 4. Discussion

Our previous studies have shown the feasibility of producing an apatite-like mineral layer on P(CL/DLLA)—BAG composite material by incubating it in SBF [9, 16]. Such biomimetic treatment is hypothesized to enhance osteogenic response to the material, as only a small portion of BAG granules in untreated composites are directly exposed to the surrounding cells or tissues creating an osteoconductive surface. The current study specifically aimed at evaluating the role of SBF incubation and subsequent calcium phosphate deposition on the osteogenic response of mesenchymal stem cells and can be seen as the first step to explore if biomimetic surface modification indeed could enhance the clinical potential of composite membranes or other tissue regeneration devices.

The beneficial effect of bioceramic filler was demonstrated by the early mineralization of all composites, and osteogenic cell response seemed to be further enhanced by a short SBF treatment. No additional benefit was found with a longer treatment time, even though the composite surface became fully covered with mineral. This was a new finding as studies involving biomimetic calcium phosphate coatings on synthetic polymers are mostly performed with uniform mineral layers [17, 18]. The adhesion of biomimetic mineral to the underlying polymer surface is, however, relatively weak and delamination may occur under physiological loads [19].

SBF treatment can have multiple roles in modifying the behavior of polymer-bioceramic composites. The growing mineral layer on specimen surfaces also increases nano- and microscale roughness [20], which in turn can enhance cell adhesion and subsequent osteogenic differentiation in culture [21, 22] and bone formation* in vivo* [23, 24]. The surface structure and chemical composition, however, are not the only factors that affect cellular responses to bioactive materials. Recent literature has demonstrated an important role of bioceramic dissolution products not only on the direct osteogenic response [25, 26] but also on angiogenesis [27, 28], both of which are crucial for successful bone regeneration. Clear decrease in the reactivity of SBF-treated composites was observed in the current study, but the concentrations of the released ions stayed at the levels deemed to be beneficial to osteogenesis.

The MSCs used in this study showed robust differentiation on all substrates. However, the cell stocks used in the two cultures differed in their osteogenic potential. More mature osteoprogenitor population was found in the first culture, as the initial stock had higher BSP expression level, ALP activity increased sooner, and mineralization started earlier than in the second culture. More reproducible cell response might have been obtained using inbred rat strain and preculturing the cells in the presence of dexamethasone, whereas the current protocol emphasizes the robustness of the material effects.

The composite examined in this study was originally developed as an injectable bone filler material, and it has been shown to enhance* in vivo* bone response in comparison to neat polymer [29, 30]. The thermoplastic nature of the polymer allows melt processing of the composite into prefabricated forms such as tissue regeneration membranes. The low working temperature of this composite system can be important, as it was recently indicated that BAG granules can unintentionally accelerate the degradation of poly(alpha-hydroxyester) matrices when processed in elevated temperatures [31].

## 5. Conclusions

In summary, we demonstrated increased mineralization of MSC cultures on polymer-BAG composites. Biomimetic treatment of the composites for five days further enhanced the osteogenic phenotype of MSCs, but longer treatment time had no additional benefits. Moreover, BAG reactivity was retained in the composites, which may be beneficial in clinical environment as the soluble ions released from the material can have the potential to improve angiogenesis in the defect site.

## Figures and Tables

**Table 1:** Substrates used in the cell culture experiments.

Substrate	Composition	SBF treatment
P0	P (CL/DLLA)	None
C0	P (CL/DLLA) + 60 wt% BAG	None
C5	P (CL/DLLA) + 60 wt% BAG	5-day immersion
C14	P (CL/DLLA) + 60 wt% BAG	14-day immersion

P (CL/DLLA): poly (*ε*-caprolactone-co-D,L-lactide).

BAG: bioactive glass S53P4, granule size 90–315 *μ*m.
